# A new spline technique for the time fractional diffusion-wave equation

**DOI:** 10.1016/j.mex.2023.102007

**Published:** 2023-01-04

**Authors:** Suruchi Singh, Swarn Singh, Anu Aggarwal

**Affiliations:** a Department of Mathematics, Aditi Mahavidyalaya, University of Delhi, India; b Department of Mathematics, Sri Venkateswara College, University of Delhi, India; c Department of Mathematics, Lakshmibai College, University of Delhi, India

**Keywords:** Fractional diffusion wave equation, Caputo-derivative, Cubic B spline, Collocation, Convergence, Spline Collocation Method

## Abstract

The current research article proposes an approximate solution of the fractional diffusion wave equation (FDWE) by using a new collocation method based on the cubic B-splines. The fractional derivative in the time direction is considered in Caputo form. The theoretical research of the proposed algorithm is discussed with *L*_∞_ and *H*^1^ norms. The method presented in this article is found to be of order (∆*t*^3−^*^α^* + *h*^4^). The highlights of the current technique proposed in this article are as under:

• The method is high-order collocation and uses a compact stencil. The error analysis is discussed to authenticate the order of convergence of the proposed numerical approximation.
• The comparisons of errors with the already existing methods are done and observed that our method produces more accurate results than the methods presented in the literature.

## Graphical abstract

**Figure:**
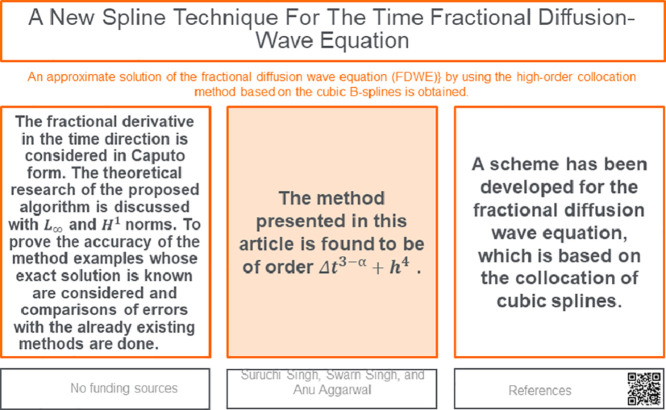

Specification table

**Table:** 

Subject area;	Mathematics
More specific subject area;	Fractional Partial Differential Equations
Name of your method;	Spline Collocation Method
Name and reference of original method;	1. D.ARCHER (1977), An o(h^4^) Cubic Spline Collocation Method for Quasilinear Parabolic Equations, SIAM Journal on Numerical Analysis, Vol.14, pp.620–637.
	2. S.SINGH, S.SINGH AND A.AGGARWAL (2017), Fourth-Order Cubic B-Spline Collocation Method For Hyperbolic Telegraph Equation, Mathematical Sciences, https://doi.org/10.1007/s40096–021–00428-y.
Resource availability;	MATLAB

## Method details

### Scientific mathematical background

In this article, we solve the fractional diffusion wave equation, numerically. This equation has been found by Einstein long back ago, but because of its viable number of applications in many fields of science, social science, and engineering problems this equation is wooing the attention of numerous scholars in the current period of time. The fractional diffusion wave equation (FDWE) has found its application in electrical networking, signal processing, electromagnetics, viscoelasticity, etc. Verily there are so many problems in science that can be modeled more accurately by fractional derivatives. By changing the time directional derivative with a fractional derivative of order *α*, in the classical diffusion equation, we can obtain a time-fractional differential equation. The FDWE is defined as:

(1) $$\frac{\;\partial^{\alpha }u}{\partial t^{\alpha }}=\nu \frac{\partial^{2}u}{\partial x^{2}}+f\left(x,t\right),$$

on a regular domain [*a,b*] × (0*,T*) subject to the I.C.’s:

(2) u(x,0)=ψ(x),a≤x≤b

and

(3) $$u_{t}\left(x,0\right)=\phi \left(x\right),a\leq x\leq b,$$

and the following Dirichlet B.C.’s:

(4) u(a,t)=0andu(b,t)=0∀t>0.

Here ν is a constant called the diffusion coefficient. The functions *ψ* and *ϕ* and their derivatives are continuous functions of

*x*. The time-fractional derivative given in Eq. (1) is the Caputo-fractional derivative, which is defined as

(5) $$\frac{\;\partial^{\alpha }u}{\partial t^{\alpha }}=\frac{1}{\Gamma \left(2-\alpha \right)}\int_{0}^{t}{\left(t-s\right)}^{-\alpha }\frac{\partial u\left(x,s\right)}{\partial s}ds,1<\alpha <2$$

with Γ denoting the gamma function and *α* is the order of the fractional time derivative. When the value of *α* = 1, Eq. (1) represents the diffusion equation and when *α* = 2, this equation represents a wave equation.

Fractional partial equations are difficult to solve analytically, because of the possession of the non-local property. Hence there is a significant demand to find new techniques or methods to solve them numerically in an efficient manner. In literature, there are plentiful methods available to solve the fractional diffusion wave equations, like the compact finite difference method, discontinuous Galerkin method, generalized differential transform method, and implicit–explicit method to name a few. An unconditionally stable fully discrete difference scheme was given by Sun and Wu in Ref. [1]. A high order scheme using Caputo fractional derivative was given by Du et al. [2]. A class of efficient finite difference methods, which are second-order convergent in space are mentioned in article [3]. Authors in the papers [[4], [5], [6], [7], [8], [9]], used finite difference method to estimate the solution of the Eq. (1) numerically. Meshless method for time fractional diffusion wave-wave equation is discussed in Ref. [10] and an advanced meshless method is given in Ref. [11]. A Fourier method for the fractional diffusion equation describing sub-diffusion is discussed by authors in Ref. [12]. Rashidinia et al. used the spectral Tau scheme in Ref. [13] to solve fractional reaction-diffusion equation. Finite element method [14] and finite volume method [15], are also used by scholars in the past few years to find the numerical solution to fractional diffusion equations. Fourth-order time-fractional problems are solved in Refs. [16,17]. In Ref. [16] authors presented fully discrete local discontinuous Galerkin method and mixed finite element method is presented in Ref. [17]. The investigations in this article are done by taking cubic B-splines as base functions and fractional derivatives in the Caputo sense. The B-spline curves are more smooth and have local support which makes them convenient to use. Authors in Refs. [18,19] handled the differential equations by using splines in their research articles. To present the high order scheme, we have used the Crank-Nicolson method for time direction in the present article.

The layout of the current article is as along the lines: Firstly, we briefly introduce the construction of spline bases and present the high order accurate scheme based on cubic splines. Then we discuss the convergence analysis of the present scheme. To prove the preciseness of our scheme, we examine some numerical examples. Lastly, an outline of all the work done in this paper is mentioned.

### Cubic B-spline technique

We first partition our domain [a,b] uniformly by taking a step size $h=\frac{b-a}{M}.\;$Let $a\;=\;x_{0}<x_{1}\cdots <x_{i-1}<x_{i}<\cdots .<x_{M}=b$ be the knots, where $x_{i+1}-x_{i}=h,\;i=0,1,2,\cdots .,M-1.$ Consider two ghost knots $x_{-1}=a-h$ and $x_{M+1}=b+h$. We define our cubic splines functions $g_{-1\;}\left(x\right),\;g_{0\;}\left(x\right),\ldots .g_{M+1\;}\left(x\right)\;$as [20]

(6) $$g_{i\;}\left(x\right)=\frac{1}{6h^{3}}\left\{ \begin{array}{cc} {\left(x-x_{i-2}\right)}^{3}, & x\;\epsilon \;\left[x_{i-2},x_{i-1}\right]\;\\ \left(h^{3}+\;\left(x-x_{i-1}\right)h^{2}+\;{\left(x-x_{i-1}\right)}^{2}h+\;{\left(x-x_{i-1}\right)}^{3}\right), & x\;\epsilon \;\left[x_{i-1},x_{i}\right]\;\\ \left(h^{3}+\;\left(x_{i+1}-x\right)h^{2}+\;{\left(x_{i+1}-x\right)}^{2}h+\;{\left(x_{i+1}-x\right)}^{3}\right), & x\;\epsilon \;\left[x_{i},x_{i+1}\right]\;\\ {\left(x_{i+2}-x\right)}^{3}, & x\;\epsilon \;\left[x_{i+1},x_{i+2}\right]\;\\ 0, & otherwise. \end{array}\right.$$

The space of all cubic spline functions defined over the interval [*a,b*], has a basis as $\left\{g_{-1},\;g_{0},\cdots ,g_{M+1}\right\}$. For

*i*= −1to*M* + 1, we list the values of the functions $\left\{g_{-1},\;g_{0},\cdots ,g_{M+1}\right\}$ and their first and second order derivatives w.r.t *x*, at the knots in the Tables 1, 2, and 3, respectively.

**Table 1:** Values of the basic functions $g_{i\;}\left(x\right)$ at the knots.

	*x*_-1_	*x*_0_	*x*_1_	*x*_2_	*x*_3_	.......	*x_M-1_*	x_M_

$g_{-1\;}\left(x\right)$	$\frac{2}{3}$	$\frac{1}{6}$	0	0	0	0	0	0
$g_{0\;}\left(x\right)$	$\frac{1}{6}$	$\frac{2}{3}$	$\frac{1}{6}$	0	0	0	0	0
$g_{1\;}\left(x\right)$	0	$\frac{1}{6}$	$\frac{2}{3}$	$\frac{1}{6}$	0	0	0	0
$g_{2\;}\left(x\right)$	0	0	$\frac{1}{6}$	$\frac{2}{3}$	$\frac{1}{6}$	0	0	0
—-	—-	—	—	—	—	—-	—-	—
$g_{M\;}\left(x\right)$	0	0	0	0	0	$\frac{1}{6}$	$\frac{2}{3}$	$\frac{1}{6}$
$g_{M+1\;}\left(x\right)$	0	0	0	0	0	0	$\;\frac{1}{6}$	$\;\frac{2}{3}$

**Table 2:** Values of the first derivative $g_{i}$ ‘(x) of the basic function at the knots.

	*x* _−_ _1_	*x*_0_	*x*_1_	*x*_2_	*x*_3_	.......	x_M-1_	*x_M_*

${g^{\prime }}_{-1}\left(x\right)$	0	$\frac{-1}{2h}$	0	0	0	0	0	0
${g^{\prime }}_{0}\left(x\right)$	$\frac{1}{2h}$	0	$\frac{-1}{2h}$	0	0	0	0	0
${g^{\prime }}_{1}\left(x\right)$	0	$\frac{1}{2h}$	0	$-\frac{1}{2h}$	0	0	0	0
${g^{\prime }}_{2}\left(x\right)$	0	0	$\frac{1}{2h}$	0	$\frac{-1}{2h}$	0	0	0
—-	—-	—-	—	–	—	—-	—-	—
${g^{\prime }}_{M\;}\left(x\right)$	0	0	0	0	0	$\frac{1}{2h}$	0	$\frac{-1}{2h}$
${g^{\prime }}_{M+1}\left(x\right)$	0	0	0	0	0	0	$\frac{1}{2h}$	0

**Table 3:** Values of the second derivative ${g^{\prime \prime }}_{i}\left(x\right)$ of the basic function at the knots.

	*x* − 1	*x*_0_	*x*_1_	*x*_2_	*x*_3_	.......	*x_M-1_*	*x_M_*

${g^{\prime \prime }}_{-1}\left(x\right)$	$\frac{-2}{h^{2}}$	$\frac{1}{h^{2}}$	0	0	0	0	0	0
${g^{\prime \prime }}_{0}\left(x\right)$	$\frac{1}{h^{2}}$	$\frac{-2}{h^{2}}$	$\frac{1}{h^{2}}$	0	0	0	0	0
${g^{\prime \prime }}_{1}\left(x\right)$	0	$\frac{1}{h^{2}}$	$\frac{-2}{h^{2}}$	$\frac{1}{h^{2}}$	0	0	0	0
${g^{\prime \prime }}_{2}\left(x\right)$	0	0	$\frac{1}{h^{2}}$	$\frac{-2}{h^{2}}$	$\frac{1}{h^{2}}$	0	0	0
—-	—-	—	—	—	—	—-	—-	—
${g^{\prime \prime }}_{M}\left(x\right)$	0	0	0	0	0	$\frac{1}{h^{2}}$	$\frac{-2}{h^{2}}$	$\frac{1}{h^{2}}$
${g^{\prime \prime }}_{M+1}\left(x\right)1$	0	0	0	0	0	0	$\frac{1}{h^{2}}$	$\frac{-2}{h^{2}}$

Let A be a cubic spline approximation to the solution. Then A can be written as the linear combination of basis functions $g_{i}^{\prime }$s for some time-dependent parameters *ρ_i_*(*t*)’s. Thus, the values of the function A and its derivatives *D_x_*A, *D_x_*^2^A at grid point (*x_i_,t*) are obtained respectively, as

$$A\left(x_{i},t\right)=\sum_{l=-1}^{M+1}\;\rho_{l}\left(t\right)g_{l}\left(x_{i}\right)=\;\frac{\;\rho_{i-1}\left(t\right)+4\;\rho_{i}\left(t\right)+\;\rho_{i+1}\left(t\right)}{6}$$

(7) $$D_{x}A\left(x_{i},t\right)=\sum_{l=-1}^{M+1}\;\rho_{l}\left(t\right)g_{l}^{{}^{\prime }}\left(x_{i}\right)=\;\frac{\;\rho_{i+1}\left(t\right)-\;\rho_{i-1}\left(t\right)}{2h}$$

$$D_{x}^{2}A\left(x_{i},t\right)=\sum_{l=-1}^{M+1}\;\rho_{l}\left(t\right){g^{\prime \prime }}_{l}\left(x_{i}\right)=\;\frac{\;\rho_{i-1}\left(t\right)-2\;\rho_{i}\left(t\right)+\;\rho_{i+1}\left(t\right)}{h^{2}}$$

In view of Eq. (7), it can be postulated that, to find numerical solution A for the differential Eq. (1), we need to determine the value of $\rho i^{\prime }s\;$for *i* = 0 to *M*. The value of $\rho_{-l}\;$and $\rho_{M+l}$ can be determined from the bc's.

We will use the Crank–Nicolson method for the discretization of the differential Eq. (1) in the time direction. Let ∆*t* denotes the step size in time direction and *t_j_*= *j∆t, j* = 1*,*2*,*3*,…,J*. Henceforth $\;A_{i}^{j}\;$denotes the value of the function A defined at the grid point $(x_{i}$, $t_{j})$ . A conventional cubic B-spline collocation scheme to solve the fractional differential Eq. (1) using the Caputo-fractional derivative (5) is given by

(8) $$\frac{1}{\Delta t\Gamma \left(\alpha -a\right)}\left(a_{0}\;\delta_{t}A_{i}^{j}-\sum_{k=2}^{\dot{j}}\left(a_{j-k}-a_{j-k+1}\right)\delta_{l}A_{i}^{k}-a_{j}\phi_{i}\right)=\nu \frac{D_{x}^{2}A_{i}^{j+1}+D_{x}^{2}A_{i}^{j}}{2}+\frac{f_{i}^{j+1}+f_{\dot{i}}^{j}}{2}$$

where,

(9) $$a_{j=}\int_{t_{j}}^{t_{j+1}}\frac{1}{t^{\alpha -1}}dt=\frac{1}{2-\alpha }\left\{{\left(t_{j+1}\right)}^{2-a}-{\left(t_{j}\right)}^{2-a}\right\}=\frac{\Delta t^{2-\alpha }}{2-\alpha }\left\{{\left(j+1\right)}^{2-a}\;-\;j^{2-a}\right\},\;$$

and

(10) $$\delta_{t}A_{i}^{j}=\frac{A_{i}^{j+1}-\;A_{i}^{j}}{\Delta t}.$$

For *j* ≥ 0, the *a_j_*’s defined above satisfy the following:

1. $a_{0}=\frac{\Delta t^{2-\alpha }}{2-\alpha }$
2. *a_j_ > a_j_*_+1_ , *j* ≥ 0,
3. For *j* ≥ 2, we have $\sum_{k=1}^{j-1}\left(a_{j-k-1\;}-\;a_{j-k}\right)=a_{0}\;-a_{j-1}$

The scheme presented by the Eq. (8) is of order *O*(∆*t*^3−^*^α^* + *h*^2^).

So, in an attempt to develop a high-order scheme, we did some modulations in Eq. (8). The new modified scheme is presented as

(11) $$\frac{1}{\Delta t\Gamma \left(2-\alpha \right)}\left(1-\frac{h^{2}}{12}D_{x}^{2}\right)L_{t}A_{j}^{i}=\nu \frac{D_{x}^{2}A_{i}^{j+1}+D_{x}^{2}A_{i}^{j}}{2}+F_{i}^{j}$$

where,

(12) $$L_{t}A_{j}^{i}=\;\left(a_{0}\;\delta_{t}A_{i}^{j}-\sum_{k=2}^{\dot{j}}\left(a_{j-k}-a_{j-k+1}\right)\delta_{l}A_{i}^{k}-a_{j}\phi_{i}\right)$$

and

(13) $$F_{i}^{j}=\;\frac{f_{i}^{j+1}+f_{\dot{i}}^{j}}{2}-\frac{h^{2}}{12}\frac{f_{xx_{i}}^{j+1}+f_{xx_{i}}^{j}}{2}$$

for *i* = 0*,*1*,*···,*M*. The scheme given by the Eq. (11) is of order *O*(∆*t*^3−^*^α^* + *h*^4^). Varying *i* = 1 to *M* in Eq. (11) will result in a system of equations, which can be solved to find the unknowns *ρ_i_*’s at time *t_j_*_+1_ and hence the approximation to the solution at time *t*.

### Convergence analysis of the scheme

This segment of the article discusses the convergence analysis of modified scheme (11).

Let us denote by *H*^1^, the Sobolev space defined on the interval [*a,b*] with the norm $\;∥u∥{\;}_{H_{0}^{1}}$ = $∥u^{\prime }∥\;$where, $∥u^{\prime }∥\;$denotes the *L*^2^ norm.

We also define a semi norm on the space *H*^1^ as,

$${\left|u\right|}^{2}=\langle u,u\rangle .$$

where, $\langle u,v\rangle =\frac{1}{N+1}\sum_{i=0}^{N}u_{i}v_{i}.$

The proof of the following lemma can be found in Ref. [21]:

**Lemma 1.***If*v*be a cubic spline function defined over the domain* [*a,b*] *and* v ∈ *H*^1^*, then*

$$\langle -v^{\prime \prime },v\rangle \geq C∥v\;{{∥}_{H^{1}}}^{2},$$

*where C is a constant which is independent of the step size h.*

**Lemma 2**. *Let a_i_’s be as defined in*Eq. (9)*, then for any functions G* = {*G*_1_*,G*_2_*,G*_3_*,*···,*G_J_*} *and ϕ, we have*

$$\sum_{j=1}^{J}\left[a_{0}G_{j}-\sum_{k=2}^{j-1}\left(a_{j-k-1}-\;a_{j-k}\right)G_{k}-a_{j-1}\phi \right]G_{j}\geq \frac{t_{J}^{1-\alpha }}{2}\Delta t\;\sum_{j=1}^{J}G_{j}^{2}-\frac{t_{J}^{2-\alpha }}{2\left(2-\alpha \right)}\phi^{2}$$

Theorem 1 Let Abe the solution of the differential Eq. (1), which satisfy Eq. (11). Then for J ≥ 1, we have

(14) $${∥A^{J+1}∥}_{H^{1}}^{2}\leq \frac{t_{J+1}^{2-\alpha }}{v\left(2-\alpha \right)\Gamma \left(2-\alpha \right)}{∥\left(1-\frac{h^{2}}{12}D_{x}^{2}\right)\phi ∥}^{2}+\frac{t_{J+1}^{\alpha -1}}{v}\Gamma \left(2-\alpha \right)\Delta t\sum_{j=1}^{J+1}{∥F^{j}∥}^{2}$$

**Proof 1**. *Taking the inner product on both sides of the*Eq. (11)*for each*i*and j with v, we have*

(15) $$\frac{1}{\;\Delta t\Gamma \left(2-\alpha \right)}\langle \left(1-\frac{h^{2}}{12}D_{x}^{2}\right)L_{t}A_{j}^{i},v\;\rangle \;=\nu \langle \frac{D_{x}^{2}A_{i}^{j+1}+D_{x}^{2}A_{i}^{j}\;}{2},v\;\rangle \;+\;\langle F_{j}^{i},v\rangle$$

*Summing up the*Eq. (15)*, from j* = 1 *to J* + 1*, we get*

(16) $$\frac{1}{\Delta t\Gamma \left(2-\alpha \right)}\sum_{j=1}^{J+1}\langle \left(1-\frac{h^{2}}{12}D_{x}^{2}\right)L_{t}A_{j}^{i},v\rangle \;=\nu \;\sum_{j=1}^{J+1}\langle \frac{D_{x}^{2}A_{i}^{j+1}+D_{x}^{2}A_{i}^{j}\;}{2},v\rangle \;+\sum_{j=1}^{J+1}\langle \;F_{j}^{i},v\rangle$$

*We choose* $v=\left(1-\frac{h^{2}}{12}D_{x}^{2}\right)\delta_{t}A_{j}^{i}\;$*in the* Eq. (15)*. After using the lemma 2, we get*

$$\sum_{j=1}^{J+1}\langle \left(1-\frac{h^{2}}{12}D_{x}^{2}\right)L_{t}A_{i}^{j},v\rangle$$

$$\;=\sum_{j=1}^{J+1}\langle \left(1-\frac{h^{2}}{12}D_{x}^{2}\right)L_{t}A_{i}^{j},\;\left(1-\frac{h^{2}}{12}D_{x}^{2}\right)\delta_{t}A_{i}^{j}\;\rangle$$

$$\;=\sum_{j=1}^{J+1}\langle a_{0}\left(1-\frac{h^{2}}{12}D_{x}^{2}\right)\delta_{t}A_{i}^{j}-\sum_{k=2}^{j-1}\left(1-\frac{h^{2}}{12}D_{x}^{2}\right)\left(a_{j-k-1\;}-\;a_{j-k}\right)\delta_{t}A_{i}^{k}-a_{j-1\;}\left(1-\frac{h^{2}}{12}D_{x}^{2}\right)\phi_{i}),\;\left(1-\frac{h^{2}}{12}D_{x}^{2}\right)\delta_{t}A_{i}^{j}\;\rangle$$

(17) $$\;\geq \frac{t_{J+1}^{1-\alpha }}{2}\Delta t\sum_{j=1}^{J+1}{\left[\left(1-\frac{h^{2}}{12}D_{x}^{2}\right)\delta_{t}A_{i}^{j}\right]}^{2}-\frac{t_{J+1}^{2-\alpha }}{2\left(2-\alpha \right)}{\left[\left(1-\frac{h^{2}}{12}D_{x}^{2}\right)\phi_{i}\right]}^{2}$$

*for each* i*. From* Eq. (17), *we obtain*

(18) $$\begin{array}{ccc} & & \sum_{j=1}^{J+1}\langle \left(1-\frac{h^{2}}{12}D_{x}^{2}\right)L_{t}A^{j},\;\left(1-\frac{h^{2}}{12}D_{x}^{2}\right)\delta_{t}A^{j}\;\rangle \\ & & \;=h\sum_{j=1}^{J+1}\sum_{i=1}^{M}\langle \left(1-\frac{h^{2}}{12}D_{x}^{2}\right)L_{t}A_{i}^{j},\;\left(1-\frac{h^{2}}{12}D_{x}^{2}\right)\delta_{t}A_{i}^{j}\;\rangle \\ & & \;\geq \frac{t_{J+1}^{1-\alpha }}{2}\Delta t\sum_{j=1}^{J+1}h\sum_{i=1}^{M}{\left[\left(1-\frac{h^{2}}{12}D_{x}^{2}\right)\delta_{t}A_{i}^{j}\right]}^{2}-\;\frac{t_{J+1}^{2-\alpha }h}{\left(2-\alpha \right)}\sum_{i=1}^{M}{\left[\left(1-\frac{h^{2}}{12}D_{x}^{2}\right)\phi_{i}\right]}^{2}\\ & & \;=\frac{t_{J+1}^{1-\alpha }}{2}\Delta t\;\sum_{j=1}^{J+1}{∥\left(1-\frac{h^{2}}{12}D_{x}^{2}\right)\delta_{t}A^{j})∥}^{2}-\;\frac{t_{J+1}^{2-\alpha }}{2\left(2-\alpha \right)}{∥\left(1-\frac{h^{2}}{12}D_{x}^{2}\right)\phi ∥}^{2} \end{array}$$

*Now, let us consider*

(19) $$\begin{array}{ccc} & & \langle D_{x}^{2}A^{j+1}+D_{x}^{2}A^{j},\;\;\left(1-\frac{h^{2}}{12}D_{x}^{2}\right)\delta_{t}A^{j}\rangle \\ & & \;=\delta_{t}\langle D_{x}^{2}A^{j},A^{j}\rangle -\frac{h^{2}}{12}\delta_{t}\langle D_{x}^{2}A^{j},D_{x}^{2}A^{j}\rangle \\ & & \;=\delta_{t}\langle D_{x}^{2}A^{j},A^{j}\rangle -\frac{h^{2}}{12}\delta_{t}{\left|D_{x}^{2}A^{j}\right|}^{2} \end{array}$$

*From lemma 1 and* (19)*, we can obtain that*

(20) $$\langle D_{x}^{2}A^{j+1}+D_{x}^{2}A^{j},\;\left(1-\frac{h^{2}}{12}D_{x}^{2}\right)\delta_{t}A^{j}\rangle \;\leq \;-\delta_{t}{∥A^{j}∥}_{H^{1}}^{2}-\frac{h^{2}}{12}\delta_{t}{\left|D_{x}^{2}A^{j}\right|}^{2}$$

*Using Young's inequality, we also have the following inequality:*

(21) $$\left|\langle F_{i}^{j},\;\left(1-\frac{h^{2}}{12}D_{x}^{2}\right)\delta_{t}A_{i}^{j}\rangle \right|\leq \;\frac{t_{J+1}^{\alpha -1}\Gamma \left(2-\alpha \right)}{2}{\left(F_{i}^{j}\right)}^{2}+\;\frac{t_{J+1}^{1-\alpha }}{2\Gamma \left(2-\alpha \right)}{\left[\left(1-\frac{h^{2}}{12}D_{x}^{2}\right)\delta_{t}A_{i}^{j}\right]}^{2}$$

*which in turn implies*

(22) $$\left|\langle F_{i}^{j},\;\left(1-\frac{h^{2}}{12}D_{x}^{2}\right)\delta_{t}A^{j}\rangle \right|\leq \;\frac{t_{J+1}^{\alpha -1}\Gamma \left(2-\alpha \right)}{2}{∥F^{j}∥}^{2}+\frac{t_{J+1}^{1-\alpha }}{2\Gamma \left(2-\alpha \right)}{∥\left(1-\frac{h^{2}}{12}D_{x}^{2}\right)\delta_{t}A^{j}∥}^{2}.$$

*From the*Eq. (16) and *the inequalities* (18)–(22)*, we obtain*

(23) $$\begin{array}{ccc} & & \frac{\nu }{2}\sum_{j=1}^{J+1}\left[\delta_{t}{∥A^{j}∥}_{H^{1}}^{2}+\frac{h^{2}}{12}\delta_{t}{|D_{x}^{2}A^{j}|}^{2}\right]+\;\frac{t_{J+1}^{1-\alpha }}{2\Gamma \left(2-\alpha \right)}\sum_{j=1}^{J+1}{∥\left(1-\frac{h^{2}}{12}D_{x}^{2}\right)\delta_{t}A^{j}∥}^{2}-\frac{t_{J+1}^{2-\alpha }}{2\left(2-\alpha \right)\Gamma \left(2-\alpha \right)}{∥\left(1-\frac{h^{2}}{12}D_{x}^{2}\right)\phi ∥}^{2}\\ & & \;\leq \frac{1}{2}\sum_{j=1}^{J+1}t_{J+1}^{\alpha -1}\;\Gamma \left(2-\alpha \right){∥F^{j}∥}^{2}+\frac{t_{J+1}^{1-\alpha }}{2\Gamma \left(2-\alpha \right)}\sum_{j=1}^{J+1}{∥\left(1-\frac{h^{2}}{12}D_{x}^{2}\right)\delta_{t}A^{j}∥}^{2}. \end{array}$$

*Thus, we get*

(24) $$∥A^{j+1}{∥}_{H^{1}}^{2}\leq \frac{t_{J+1}^{2-\alpha }}{\nu \left(2-\alpha \right)\Gamma \left(2-\alpha \right)}{∥\left(1-\frac{h^{2}}{12}D_{x}^{2}\right)\phi ∥}^{2}+\;\frac{t_{J+1}^{\alpha -1}\Gamma \left(2-\alpha \right)\Delta t}{\nu }\sum_{j=1}^{J+1}{∥F^{j}∥}^{2}.$$

*This is true for all* J≥*0.*

Once, we obtain the $\rho_{i}$(0) values of the unknown parameters from the initial and bc's, we can find the values of *ρ_i_*(*t*)’s at the subsequent time levels using the scheme given by the Eq. (11). Hence the approximate solution and its derivative at time *t* can be obtained by putting the values of *ρ_i_*(*t*)’s in the Eq. (7).

## Numerical experiments

In the present section, we have considered a couple of examples taken from the literature to testify to the proficiency and veracity of the proposed method. We have solved those problems whose exact solutions are given. The execution of all the computational work has been done using MATLAB. We calculate the maximum absolute error and the order of convergence between the exact and the numerical solution of the differential equation. The maximum absolute error is obtained by the formula:

$$\text{error}=\max_{i}\left|\text{ui}-\;Ai\right|$$

The order of convergence for the given method is calculated using the formula:

$$\mathrm{order}\;\mathrm{of}\;\mathrm{conv}\mathrm{erge}\mathrm{nce}=\frac{\text{log}\left(\mathrm{error}\left(h\right)\right)\text{-log}\left(\mathrm{error}\left(h/2\right)\right)}{\log2}$$

Example 1. Consider the time-fractional diffusion equation

$$\frac{\;\partial^{\alpha }u}{\partial t^{\alpha }}=\frac{\partial^{2}u}{\partial t^{2}}+f,\;1\;\leq \;\alpha \;\leq \;2,x,t\in \left[0,1\right],$$

with I.C.’s

$$u\left(x,0\right)=0,{\text{u}}_{t}\left(x,0\right)=0,0\leq x\leq 1.$$

The B.C.’s are

u(0,t)=0,u(1,t)=0,0≤t≤1.

The exact solution of this problem is $u\left(x,t\right)=t^{3}\sin\left(\pi x\right),$

In Table 4, maximum absolute errors and their orders of convergence are obtained for Example 1 at *T* = 1. The errors and orders are calculated for *α* = 1*.*1 and by taking $\Delta t\propto \frac{1}{h^{2}}$. The comparisons of the errors are done with those given in the paper [5]. It is clearly evident from the Table 4, that the errors calculated by the present method are of fourth order and are much better than those listed in Ref. [5].

**Table 4:** Comparison of maximum absolute errors and their orders obtained for Example 1, for ∆*t* = 1*/h*^2^ and *α* = 1*.*1.

h	Present Method	Order	[5]		Order

1/5	4.15(−4)	—	2.30(−2)		—-
1/10	2.73(−5)	3.92	6.00(−3)		1.93
1/20	1.63(−6)	4.06	1.49(−3)		2.00
1/40	9.56(−8)	4.09	3.75(−4)		1.99
1/80	5.49(−9)	4.12	9.41(−5)		1.99

Example 2. Consider the time-fractional diffusion equation

$$\frac{\;\partial^{\alpha }u}{\partial t^{\alpha }}=\frac{\partial^{2}u}{\partial t^{2}}-2t^{2}+\frac{2t^{2-\alpha }}{\Gamma \left(3-\alpha \right)}x\left(x-1\right),1\;\leq \;\alpha \;\leq \;2,\;x,t\in \;\left[0,1\right],$$

with I.C.’s

$\;u\left(x,0\right)=0,u_{t}\left(x,0\right)=0,0\leq x\leq 1$*.* The B.C.’s are

u(0,t)=0,u(1,t)=0,0≤t≤1.

The exact solution to this problem is $\;u\left(x,t\right)=t^{2}x\left(x-1\right)$.

**Fig. 1:**
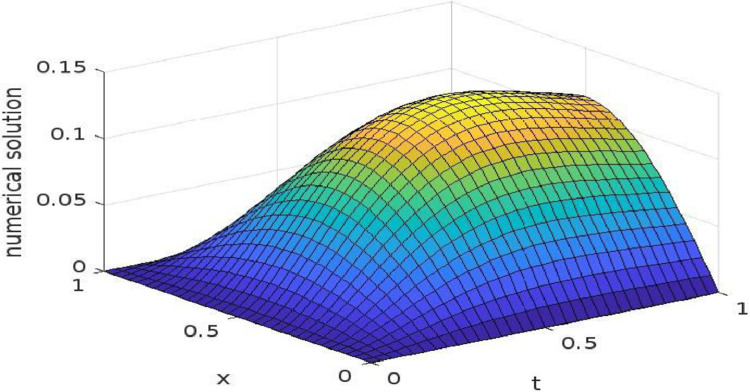
Time-space graph of numerical solution of example 2 for (*x,t*) ∈ [0*,*1].

In the following tables, we have shown the calculations of maximum absolute errors and the orders for Example 2, by taking different values of *α*. The comparisons of the results are done with those listed in Ref. [10]. Table 5, shows the errors and their order of convergence, computed for *α* = 1*.*85 and different value of *h* and ∆*t*. In Table 6, Table 7, Table 8, results are calculated by taking time step size ∆*t* = 1*/*500. Tables 6 and 7 shows the comparisons of errors and their orders with those listed in Ref. [10] by taking *α* = 1*.*5 and *α* = 1*.*25, respectively, whilst Table 8 exhibit the results for *α* = 1*.*15. From the given tables it can be easily drawn out that the method suggested in this article is of fourth order and more explicit than those given in the literature. The time-space graph of the numerical solution calculated for *α* = 1*.*85 with *h* = 1*/*15 and ∆*t* = 1*/*16 has been shown in Fig. 1.

**Table 5:** Maximum absolute errors and their orders obtained for Example 2, for *α* = 1*.*85.

h	∆*t*	Present Method	Order

1/15	1/16	3.818(−6)	—-
1/30	1/64	2.466(−7)	3.92
1/60	1/256	1.551(−8)	3.99
1/120	1/1024	9.708(−10)	4.00

**Table 6:** Comparison of maximum absolute errors and their orders obtained for Example 2, for ∆*t* = 1*/*500 and *α* = 1*.*5.

h	Present Method	Order	[10]	Order

1/4	5.277(−4)	—-	4.954(−3)	—
1/8	3.268(−5)	4.00	1.778(−3)	1.47
1/16	2.052(−6)	4.00	8.993(−4)	0.98
1/32	1.282(−7)	4.00	—	—
1/64	8.617(−9)	4.00	—	—
1/128	5.011(−10)	4.00	—	—

**Table 7:** Comparison of maximum absolute errors obtained for Example 2, for ∆*t* = 1*/*500 and *α* = 1*.*25.

h	Present Method	Order	[10]	Order

1/4	4.474(−4)	—	5.039(−3)	—
1/8	2.864(−5)	3.97	1.812(−3)	1.47
1/16	1.815(−6)	3.98	9.086(−4)	0.99
1/32	1.143(−7)	3.99	—	—
1/64	7.716(−6)	3.99	—	—
1/128	4.494(−10)	4.00	—	—

**Table 8:** Comparison of maximum absolute errors obtained for Example 2, for ∆*t* = 1*/*500 and *α* = 1*.*15.

h	Present Method	Order	[10]	Order

1/4	4.243(−4)	—	5.066(−3)	—
1/8	2.746(−5)	3.94	1.824(−3)	1.47
1/16	1.752(−6)	3.97	9.119(−4)	1.00
1/32	1.107(−7)	3.98	—	—
1/64	6.962(−9)	3.99	—	—
1/128	4.364(−10)	3.99	—	—

Example 3. Consider the time-fractional diffusion equation

$$\frac{\;\partial^{\alpha }u}{\partial t^{\alpha }}=\frac{\partial^{2}u}{\partial t^{2}}+\sin\left(\pi x\right),\;1\;\leq \;\alpha \leq 2,\;x,t\in \;\left[0,1\right],$$

with I.C.’s $u\left(x,0\right)=0,u_{t}\left(x,0\right)=0,0\leq x\leq 1$*.*

The B.C.’s are

u(0,t)=0,u(1,t)=0,0≤t≤1.

The exact solution of this problem is $u\left(x,t\right)=\;\frac{1}{\pi^{2}}[1-E_{\alpha }\left(-\pi^{2}t^{\alpha \;}\right]\text{sin}\left(\pi x\right)$, where *E_α_z* is a Mittog–Lefter function in one parameter defined by the series expansion $E_{\alpha }z=\sum_{k=0}^{\infty }\frac{z^{k}}{\Gamma (\alpha k+1)}$

If *α* = 3*/*2, then we have:

$$u\left(x,t\right)=\frac{1}{\pi^{2}}\left[\frac{1}{\sqrt{\pi }}\sum_{m=1}^{\infty }\frac{{\left(\pi^{2}t^{\alpha }\right)}^{2m-1}}{\prod_{i=1}^{3m-1}\left(i-\frac{1}{2}\right)}-\;\sum_{m=1}^{\infty }\frac{{\left(\pi^{2}t^{\alpha }\right)}^{2m}}{\prod_{i=1}^{3m-1}i}\right]\;$$

**Fig. 2:**
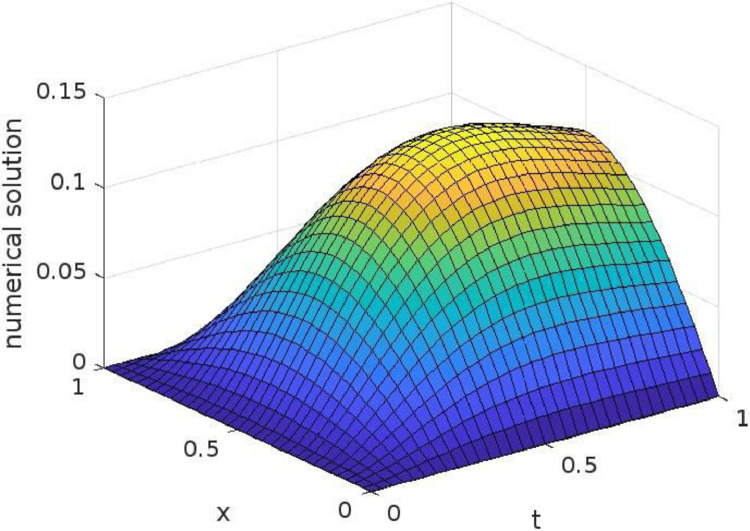
Time-space graph of numerical solution of example 3 for (*x,t*) ∈ [0*,*1].

We have shown the maximum absolute errors and their comparisons with Ref. [1] in Table 9. The results are found for *α* = 3*/*2 at *T* = 1 and we have taken *h* = ∆*t*. The results are compared with those listed in Ref. [1]. The time-space graph for the numerical solution found for *α* = 3*/*2 with *h* = ∆*t* = 3*/*2 is shown in Fig. 2. It is very clear that the results calculated by the present technique are more accurate.

**Table 9:** Comparison of maximum absolute errors obtained with *h* = ∆*t* for Example 3.

h	Present Method	[1]

1/64	1.362(−4)	4.330(−2)
1/128	4.891(−5)	4.326(−2)
1/256	1.744(−5)	4.325(−2)
1/512	6.199(−6)	4.324(−2)

## Conclusion

In this research article, we have developed a scheme for the fractional diffusion wave equation, which is based on the collocation of cubic splines. In our paper, we presented the convergence analysis of the proposed scheme. In the end, using MATLAB for computation, we obtained the errors and order of convergence of some examples and did a comparison of the results with those presented in the literature. During the course of the study, we have found that the method presented in this paper is more accurate and is of fourth order.

## CRediT authorship contribution statement

**Suruchi Singh:** Conceptualization, Methodology, Supervision, Writing – review & editing. **Swarn Singh:** Conceptualization, Formal analysis, Writing – original draft, Software, Validation. **Anu Aggarwal:** Writing – original draft, Software, Validation.

## Declaration of Competing Interest

The authors declare that they have no known competing financial interests or personal relationships that could have appeared to influence the work reported in this paper.

## Data Availability

No data was used for the research described in the article.
